# Diagnosis and treatment of traumatic Descemet’s membrane detachment: A case series

**DOI:** 10.1097/MD.0000000000034121

**Published:** 2023-06-23

**Authors:** Zhao Li, Wen Gao, Yongli Yang, Weilin Liang

**Affiliations:** a Ophthalmic Center, General Hospital of Xinjiang Military Region, Urumqi, China; b Ophthalmic Center, 474 Hospital of Xinjiang, Urumqi, China; c PLA General Hospital Jingxi Medical Area, Beijing, China.

**Keywords:** case report, congenital glaucoma, Descemet’s membrane, Habb’s striae, intraocular surgery

## Abstract

The Descemet layer is a dense layer of tissue that does not detach under normal circumstances. Descemet layer detachment may occur after intraocular surgery, but the Descemet layer spontaneously detached after trauma in this child, which is relatively rare. After looking for the cause, we found that the child was diagnosed with congenital glaucoma, and the trauma induced the Descemet’s membrane detachment.

**Patient concerns:**

The parents of the patient expected the child to recover the normal shape of the cornea as soon as possible, improve vision, and solve the problem of congenital glaucoma.

**Diagnoses:**

The patient was diagnosed with Descemet's membrane detachment of the left eye and congenital glaucoma in both eyes.

**Interventions:**

During operation, inflation gas is injected into the anterior chamber, the Descemet’s membrane is reset, and glaucoma surgery is performed.

**Outcomes:**

The Descemet’s membrane in the child’s eye was reset, and after glaucoma surgery, the intraocular pressure of the child was normal.

**Lessons:**

The analysis of the disease is not only to solve the problems seen but also to deeply analyze the internal causes and pathological changes in combination with the symptoms and signs, so as to discover the essence of the problem and solve the fundamental problem of the patient.

## 1. Introduction

The Descemet membrane is a basement membrane for endothelial cells, which migrate into the trabecular tissue. The membrane has a certain elasticity and can be regenerated after injury.^[1]^ Descemet’s membrane detachment (DMD) refers to the separation of the Descemet’s membrane and the endothelial cell layer from the corneal stroma.^[2]^ In ophthalmic clinical practice, DMD is mostly reported after cataract phacoemulsification, Extracapsular Cataract Extraction (ECCE), and glaucoma trabeculectomy.^[3]^ therefore, a history of intraocular surgery is an indication for most DMD cases.^[4]^ In this study, the patient had no history of intraocular surgery and was a rare case of trauma associated with DMD.

## 2. Case presentation

### 2.1. Patient information

**Figure 1.**
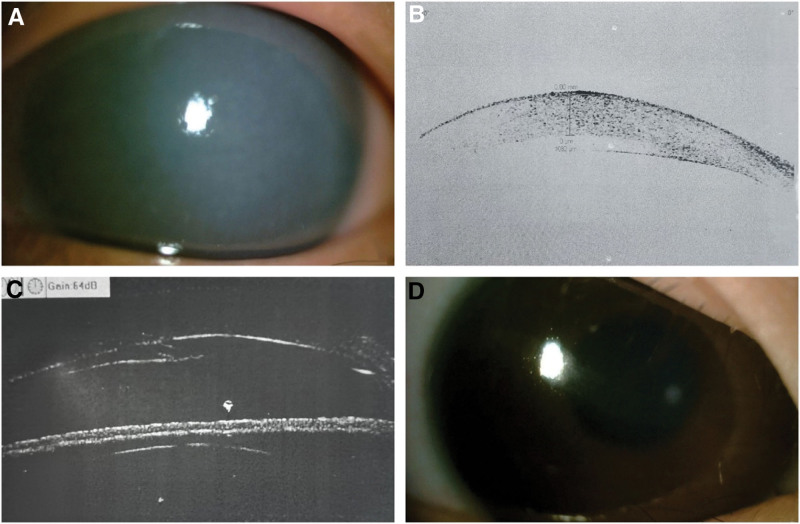
(A) Image of the right eye. (B) Anterior segment OCT revealed DMD in the left eye. (C) UBM revealed a partial detachment of the corneal endothelial layer in the left eye. (D) Haab’s striae in the right eye. DMD = Descemet’s membrane detachment, OCT = optical coherence tomography, UBM = ultrasound biomicroscope.

The patient, a 4-year-old female, visited the outpatient clinic reporting a “whitening of the cornea in the left eye, which occurred 5 days prior to the visit to our clinic,” and 2 days after an accidental fall of the child Due to the lack of any other symptoms or discomfort, not treatment was applied in the first 2 days after the accident. On the third day, the patient’s left eye manifested a gradual whitening and was originally diagnosed by a specialist with left eye keratitis, and anti-inflammatory and antiviral treatment was prescribed. The symptoms insisted and the patient continued to receive anti-inflammatory and corneal nutritional treatment. On day 7 after the accident, the symptoms worsened significantly, and the patient was sent to our hospital for further treatment. Visual inspection showed that the right eye was 0.1 and the left eye had poor coordination, and the child was not able to cooperate during the intraocular pressure examination. The examination revealed a significant edema in the left cornea, and the upper temporal corneal Descemet’s membrane showed a sheet-like, arc-shaped translucent area. The iris texture appeared blurred and visible, but no other tissue was visible (Fig. 1A). Both the lens and the cornea of the right eye appeared transparent and the endothelium showed linear texture. The depth of the anterior chamber was measured at roughly 2.5 corneal thickness, the texture of the iris appeared clear, the color of the optic disc was slightly lighter than normal in the fundus, cup disc ratio was 0.6 and no retinal anomalies were observed. Past history: The patient had no history of eye surgery. One month prior to the ocular accident, however, she suffered from a fractured right wrist and was immobilized in a plaster cast.

The ocular examination showed that the initial keratitis diagnosis was inaccurate. The cause of the macroscopic “whitening of the cornea” was likely due to the abnormal function of the endothelial “pump.” We performed an optical coherence tomography (OCT) examination to check for corneal edema, and evaluate the health of Descemet’s membrane, the endothelial layer, and anterior chamber of the left eye. The OCT revealed that the left eye had corneal edema, the corneal endothelial layer was peeled off, and the central corneal thickness was 1082 µm (Fig. 1B). After confirming the diagnosis of DMD in the left eye, the patient was admitted to the hospital for further treatment.

Although DMD in the left eye was confirmed, its cause remained unclear, because the corneal tissue appeared dense, and DMD is commonly seen after cataract phacoemulsification, ECCE, or glaucoma trabeculotomy, whereas DMD caused by traumatic events is very rare. Further ophthalmological examinations were carried out after admission, to determine the etiology and possible causes of the injury,

#### 2.1.1. Results.

The OCT examination revealed that the thickness of the optic disc of the right eye was 50 µm, indicating optic nerve atrophy of the right eye. There was the fovea of the right eye, and the remaining tissue structure was intact. As for the left eye, no structure could not be seen. Visual evoked potential showed weak signal of the right eye and delayed peak time of the left eye, with acceptable amplitude. Ocular ultrasonography suggested that both eye axis were normal. Ultrasound Biomicroscope showed a partial detachment of the corneal endothelial layer of the left eye (Fig. 1C). Intraocular pressure was 45 mm Hg in the right eye, and not measurable in the left eye. A visual fields examination was not carried out, due to the inability of the patient to cooperate.

The above findings were inconsistent and the etiology of the high Intraocular Pressure and optic nerve atrophy was unclear. In order to exclude non-ocular factors, Intraocular Pressure was measured again and it was found to be 47 mm Hg in the right eye and 7 mm Hg in the left eye. Optic disk OCT was repeated with the same results. At this point, the patient’s medical history of right wrist fracture and the incidence when the patient fell on his face was reviewed. The parent stated that they thought the injuries were due to calcium deficiency. During the physical assessment of the eye, we concluded that the linear texture of the cornea of the right eye was Haab striae (Fig. 1D). Based on this, it was believed that the child had congenital glaucoma in both eyes, which resulted in visual loss due to the lack of treatment, causing the frequent falls. Long-term high intraocular pressure caused an irreversible damage to the optic nerve and cornea. When the intraocular pressure is high, the edema of the cornea and the Descemet’s membrane are relatively loose. Studies have shown that changes in the function of the corneal endothelial layer can reduce adhesion between Descemet’s membrane and the stroma. Furthermore, studies reported that glaucoma can cause corneal endothelial dystrophy and changes in the Descemet’s membrane. The frequent falling caused DMD in the rupture zone, resulting in corneal stromal edema, which was manifested by the “whitening of the left cornea.” Hence, we identified the pathophysiological process of Descemet’s membrane rupture due to congenital glaucoma in both eyes and DMD in the left eye due to trauma and the frequent falls.

### 2.2. Treatment

Gas injection into the anterior chamber is often used to repositioning the Descemet’s membrane. However, due to the patient’s congenital glaucoma and the long-standing DMD, a number of issues arise. The poor corneal condition demands extensive intraocular pressure control. Moreover, the location of the main incision is very important, it needs to not only meet the re-positioning requirements, but also to avoid re-injury of the membrane.

**Figure 2.**
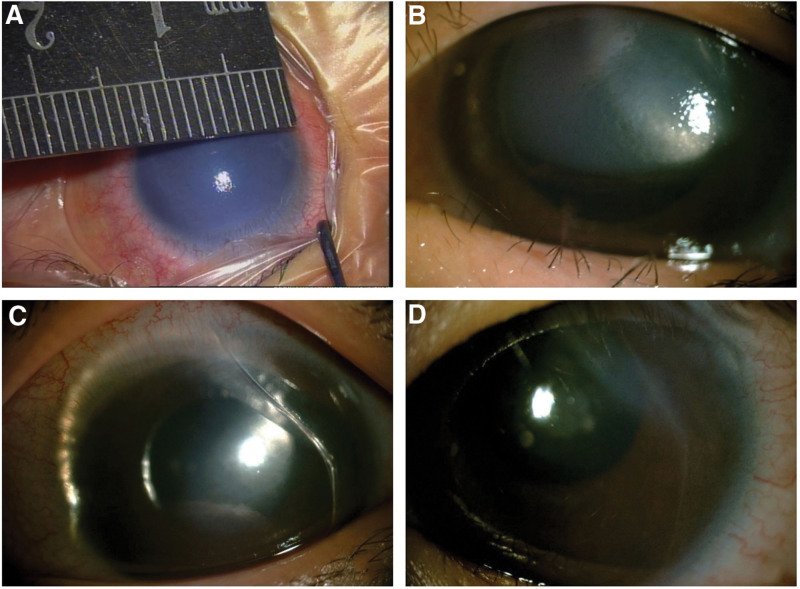
(A) Intraoperative corneal measurement. (B) Corneal edema relieved on the first day after the surgery. (C) Corneal edema relieved further on the sixth day after the surgery. (D) Gas was absorbed and cornea was clear on the 30th day after the surgery.

Ultimately, we decided to use a superior temporal 15° angle to make an incision through the scleral limbus. Both corneas was measured during surgery, at 14 mm (Fig. 2A). Perfluoropropane (C3F8) with a concentration of 14% was injected from the arc translucent area above the temporal area to the anterior chamber. The cornea appeared slightly transparent. The gas mixture of C3F8 and air was adjusted to a large air bubble in the anterior chamber, the puncture port was sealed so that there was no air leakage or bleeding.

## 3. Results

On the first day after the surgery, the air bubble was in place, the corneal edema was relieved, and there was no complaints of discomfort (Fig. 2B). On the second day, the patient complained of eye pain and headache; the anterior chamber depth was found to be about 3.5 corneal thickness. In order to decrease pressure, 125 mL of mannitol was given intravenously, and intraocular pressure- lowering drugs were given to both eyes. In the following 3 days, half-dose of mannitol was given every 24 hours to lower intraocular pressure, and C3F8 gas remained at top pressure (Fig. 2C). By day 6 after the surgery, one third of the gas was absorbed, the corneal edema was relieved, and the iris texture was blurry. Trabeculectomy was scheduled for the glaucoma of the right eye, which, however, was not performed because the patient contracted influenza and developed fever and was transferred to the Children’s Hospital. At 1 month follow-up, the left eye cornea was transparent, the gas was completely absorbed, and Descemet’s membrane was repositioned (Fig. 2D). The corneal fissure was still visible, and the patient was admitted for glaucoma treatment.

## 4. Discussion and conclusions

The patient was originally examined by a physician reporting a “whitening of the cornea of the left eye.” The physician prescribed drug treatments for keratitis. After comprehensive examination in our clinic, the patient was diagnosed with DMD of the left eye. There was increased intraocular pressure and optic nerve atrophy in the right eye. After careful assessment of all symptoms, we concluded that DMD of the left eye was caused by a ruptured Descemet’s membrane as a result of congenital glaucoma in both eyes. This condition is occult to a certain extent, and it is easy to be misdiagnosed if relying solely on symptoms. Thus, it is necessary to analyze the corneal structure in detail and consult examination results. Due to our current understanding that DMD is only seen in cataract phacoemulsification, ECCE, and glaucoma trabeculectomy, the correct diagnosis required a nuanced reasoning process.^[5]^ Since corneal structure is typically tight, trauma alone is not common to cause DMD^[6]^, and thus it is necessary to look for the undelying cause. For the patient in this study, the root cause of rupture of the Descemet’s membrane was congenital glaucoma and was identified only after examinations of the healthy eye, and trauma was the cause of DMD. The patient suffered from frequent falls, which was also caused by long-term high intraocular pressure and optic nerve atrophy due to congenital glaucoma.^[7]^ Once the cause was determined, the surgical procedure was not complicated. The objective of the treatment is to reposition Descemet’s membrane through gas injection. If the treatment of the patient is further delayed and the corneal endothelial function is further damaged, the treatment outcome can vary significantly. The case study shows that children with congenital glaucoma must be treated as soon as possible^[8]^, and intraocular pressure control measures need to be taken to avoid further damage to the eye. Moreover, for the diagnosis and treatment of eye diseases, it is necessary to identify the root cause from a multi-dimensional, multi-timeline perspective, rather than just focusing on the most obvious pathology. Only through examination of the right eye, the patients full medical history, and complete physical assessment we were able to reach the final conclusion.

## Acknowledgments

The authors would like to express their gratitude to EditSprings (https://www.editsprings.cn) for the expert linguistic services provided.

## Author contributions

**Formal analysis:** Weilin Liang.

Investigation: Zhao Li, Wen Gao, Yongli Yang.

Methodology: Yongli Yang.

Project administration: Wen Gao.

Resources: Weilin Liang.

Writing – original draft: Zhao Li.

Writing – review & editing: Wen Gao.
